# Application of Gold Nanoparticle to Plasmonic Biosensors

**DOI:** 10.3390/ijms19072021

**Published:** 2018-07-11

**Authors:** Jin-Ho Lee, Hyeon-Yeol Cho, Hye Kyu Choi, Ji-Young Lee, Jeong-Woo Choi

**Affiliations:** 1 Department of Chemical and Biomolecular Engineering, Sogang University, 35 Baekbeom-ro, Mapo-gu, Seoul 04107, Korea; jino@sogang.ac.kr (J.-H.L.); yeol@sogang.ac.kr (H.-Y.C.); hyekchoi@sogang.ac.kr (H.K.C.); jylee72@sogang.ac.kr (J.-Y.L.); 2 Department of Chemistry and Chemical Biology, Rutgers, The State University of New Jersey, Piscataway, NJ 08854, USA

**Keywords:** gold nanoparticles, plasmon, biosensor, molecular diagnosis

## Abstract

Gold nanoparticles (GNPs) have been widely utilized to develop various biosensors for molecular diagnosis, as they can be easily functionalized and exhibit unique optical properties explained by plasmonic effects. These unique optical properties of GNPs allow the expression of an intense color under light that can be tuned by altering their size, shape, composition, and coupling with other plasmonic nanoparticles. Additionally, they can also enhance other optical signals, such as fluorescence and Raman scattering, making them suitable for biosensor development. In this review, we provide a detailed discussion of the currently developed biosensors based on the aforementioned unique optical features of GNPs. Mainly, we focus on four different plasmonic biosensing methods, including localized surface plasmon resonance (LSPR), surface-enhanced Raman spectroscopy (SERS), fluorescence enhancement, and quenching caused by plasmon and colorimetry changes based on the coupling of GNPs. We believe that the topics discussed here are useful and able to provide a guideline in the development of novel GNP-based biosensors in the future.

## 1. Introduction

Recent advances in nanotechnology have revealed new possibilities for the development of biosensors with improved performance based on the unique properties of nanoparticles [1]. For instance, the high surface area-to-volume ratios of nanomaterials facilitate the performance of bio/chemical analyte capturing and sensing by providing more active surface regions. More specifically, magnetic nanoparticles can be employed for the enrichment of targets analytes, and polymeric or plasmonic metal nanoparticles can be introduced for signal amplification [2,3,4].

Among these nanoparticles, plasmonic nanoparticles have gained particular interest from various scientists and engineers because of their unique physical and chemical properties. They have been widely applied in the biomedical fields for diagnostics, imaging, and therapeutics [2,5,6]. In particular, gold nanoparticles (GNPs) have drawn enormous interest, owing to their easy preparation, biocompatibility, inertness, and intense colors [6,7]. Additionally, the ease of surface modification with a wide range of biomolecules, such as oligonucleotides and proteins, through the formation of stable bonds with mercapto or amino groups to create specific/selective binding, allows for the development of new biosensor platforms with enhanced capabilities in the detection of various biological/chemical analytes [6,8,9].

One of the most prominent and useful features of GNPs for biosensor applications is their unique and tunable optical properties. In particular, localized surface plasmon resonance (LSPR) can be modulated by different sizes, shapes, and surface coupling of the nanoparticles [10,11,12,13]. Upon irradiation with light, the free electrons in the GNP are excited, simultaneously inducing collective coherent non-propagating oscillations of surface plasmons, which is known as LSPR [14]. Gold surface plasmons exhibit unique features underlying their strong absorption and scattering of light, making GNPs fascinating candidates for biological sensing and imaging probes. Briefly, the localization of the electromagnetic field results in the amplification of SPR signals and surface-enhanced Raman scattering (SERS) [1,15]. Fluorescent signals can be also quenched or enhanced due to the energy transfer between the GNP and fluorophore [16]. Additionally, a high molar extinction coefficient and significant color changes can be observed by aggregating GNPs, resulting in the development of naked-eye-based biosensors [17]. Furthermore, many studies have already revealed that GNPs are relatively nontoxic when compared to other nanoparticles, such as metal oxides or carbon-based materials in general [5,18]. This qualification of GNPs has led to the development of biosensors for in vitro/in vivo diagnosis and imaging of molecular interactions on a cellular level as well.

In this paper, we review and discuss recently developed plasmonic biosensors combined with the unique optical properties of GNPs. We provide an overview of the unique optical properties of GNPs utilized for the development of biosensors and then highlight newly developed GNP-based plasmonic biosensors. Each section individually focuses on one of the following: LSPR, SERS, plasmon-enhanced fluorescence and quenching, and colorimetry changes based on the coupling of plasmonic GNPs.

## 2. Gold Nanoparticle-Based Localized Surface Plasmon Resonance Biosensor

One of the most well-established unique optical characteristics of GNPs that is widely utilized for the development of biosensors is their localized surface plasmon resonance (LSPR) phenomenon [19,20]. When noble metals, such as gold, are exposed to light, a resonant interaction between electron-charged oscillations near the surface of the metal and the electromagnetic field of the light creates propagating surface plasmons. This unique physical property changes enormously when the size of metal becomes nanoscale relative to the bulk material, resulting in a confined and non-propagating localized surface plasmon around the nanoparticle with a specific frequency known as the LSPR [19,21]. In the particular case of GNPs, the LSPR yields exceptionally high absorption coefficients and scattering properties within the visible to near-infrared (NIR) wavelength range [22,23]. These spectral characteristics of GNPs are independent of size, shape, and the local dielectric environment, therefore, the refractive index changes induced by an adsorbate on a GNP can be used to monitor molecular binding events [12,24].

**Figure 1:**
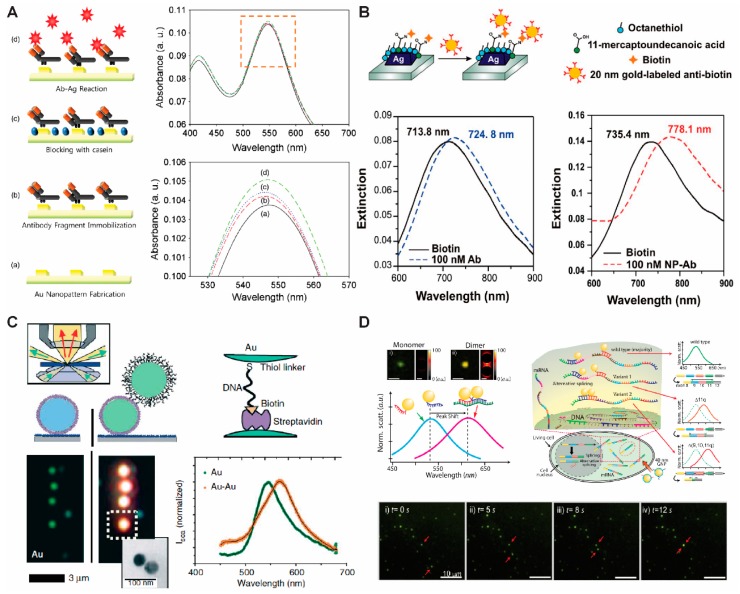
Plasmonic biosensor based on localized surface plasmon resonance (LSPR). (**A**) Molecular detection based on gold nanoparticle array platform; (**B**) signal amplification of LSPR sensor based on gold nanoparticle; (**C**) analysis of DNA length and hybridization kinetics based on dimerization of gold nanoparticles; (**D**) detection of mRNA splice variants in live cells based on dimerization of gold nanoparticles. (**A**) Figure reproduced with permission from [22], © 2013 Elsevier; (**B**) Figure reproduced with permission from [23], © 2011 American Chemical Society; (**C**) Figure reproduced with permission from [24], © 2005 Nature Publishing Group; (**D**) Figure reproduced with permission from [9], © 2014 Nature Publishing Group.

Recently, several studies on the development of biosensors based on LSPR have been reported. Lee et al. have proposed a simple electrochemical deposition technique to fabricate GNPs on a transparent indium tin oxide substrate for the detection of human immunodeficiency virus (HIV) based on the LSPR mechanism (Figure 1A) [25]. The binding of biomolecules on the GNP surface was quantitatively analyzed through absorbance changes, which are caused by the change in refractive index on the GNP surface due to specific binding occurring through immunoreactions without any labeling materials. Comparably, Van Duyne and his group used GNPs as a labeling material to improve the signal of an LSPR-based immunoassay. Anti-biotin antibodies were labeled with 20 nm GNPs and introduced to a biotin-functionalized silver nanoprism array. As a result, a 42.7 nm wavelength shift was induced, while unlabeled anti-biotin resulted in only an 11 nm redshift. (Figure 1B) [26].

As an alternative approach, instead of using array systems, single GNP spectroscopy is also utilized for biosensing by using dark-field microscopy techniques. Compared to array systems, single-nanoparticle-based LSPR biosensors have several advantages, such as higher sensitivity and smaller sample volume requirements. Alivisatos and his coworkers have used spectral changes based on the dimerization of GNPs to measure DNA length and track hybridization kinetics. The distance was determined on the basis of plasmonic coupling between two GNPs modified at two ends of a DNA strand (Figure 1C) [27]. Two GNPs were separately conjugated to each single-stranded DNA (ssDNA) end by gold–thiol interactions on one end, and streptavidin–biotin interactions on the other end. The distance between the GNPs was adjusted by controlling the length of the ssDNA and by changing the ionic strength of the buffer. The plasmon resonance (LSPR) maximum shifted to the red region at high salt concentrations (0.1 M NaCl), indicating the decreased distance between the two GNPs due to the reduced electrostatic repulsion of the particles at high ionic strength environments. Conversely, low salt concentrations (0.005 M NaCl) increased electrostatic repulsions and led to a blue shift of the LSPR maximum. Along with these results, the hybridization of complementary DNA also resulted in a significant blue shift, which is expected considering that a structural property of double-stranded DNA (dsDNA) is a stiffness greater than ssDNA, therefore allowing it to push apart two GNPs. Lee et al. also demonstrated that a combination of plasmonic dimer probes can be used to detect and image mRNA splice variants in live cells [11]. The GNPs were functionalized with oligonucleotides as a probe, and the hybridization of these probes with specific mRNA sequences led to the formation of nanoparticle dimers that exhibited distinct spectral shifts due to their plasmonic coupling. With this approach, they showed the spatial and temporal distribution of three selected splice variants of the breast cancer susceptibility gene (BRCA1) and also monitored single-copy resolutions by measuring the hybridization dynamics of nanoplasmonic GNP dimers (Figure 1D).

## 3. Gold Nanoparticle-Based Surface-Enhanced Raman Spectroscopy Biosensing

**Figure 2:**
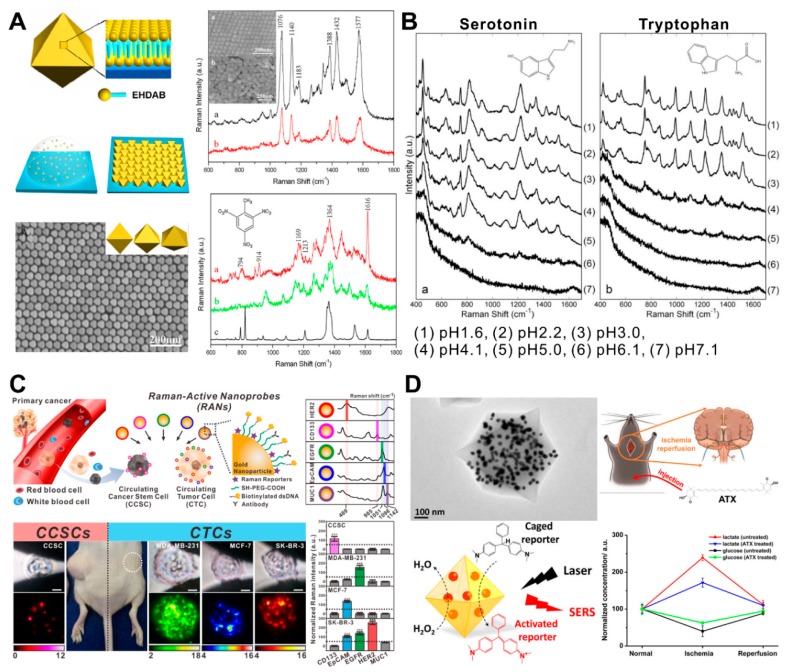
Plasmonic biosensor based on surface-enhanced Raman spectroscopy (SERS). (**A**) Highly ordered octahedral gold nanoparticle (GNP) arrays for Raman signal enhancement; (**B**) pH-dependent SERS signal differences between serotonin and tryptophan based on gold nanoparticles; (**C**) Raman barcoding-based cancer subtyping platform based on combination of Raman reporter tagged gold nanoparticles; (**D**) detection of glucose in live mouse tissue based on dimerization of gold nanoparticles. (**A**) Figure reproduced with permission from [37], © 2011 Elsevier; (**B**) Figure reproduced with permission from [38], © 2010 Society of Photo-Optical Instrumentation Engineers; (**C**) Figure reproduced with permission from [42], © 2018 Elsevier; (**D**) Figure reproduced with permission from [43], © 2017 American Chemical Society.

Raman spectroscopy is a spectroscopic technique based on the inelastic scattering of photons from the targeting molecules in the sample which reflects the specific chemical bond information. Even though Raman spectroscopy has a great potential to be applied as a biosensor by taking advantage of its specificity, the weak signal intensity is its main drawback. The LSPR phenomenon of plasmonic GNPs has been utilized to enhance the light scattering signal for biosensing applications. LSPR leads to resonant absorption or scattering of the incident light, thereby effectively coupling the incident light energy into the metal nanoparticles, including GNPs. This results in a 2–5-order magnitude enhancement of the local electromagnetic (EM) field intensity at the nanoparticle surface, which is the key to the huge enhancement in SERS [28,29]. Moreover, recent studies of SERS on metal nanoparticles demonstrated the importance of having local field “hotspots” due to surface roughness [30,31], nanogaps between aggregated plasmonic NPs [32,33], or NP-metal surfaces [34] which can induce higher EM enhancements, and the SERS contribution to such hotspots often dominates the observed response [31]. To generate homogeneous “hotspots” using GNPs, researchers have fabricated GNP arrays [35,36]. Liu and coworkers utilized a simple droplet evaporation process to assemble highly ordered octahedral GNP arrays with nanoscale interparticle gaps (Figure 2A) [37]. To form highly ordered nanogaps, which have a role as a “hotspot” for SERS, gold octahedra with an edge length of about 42 nm were used as building blocks for 2D and 3D superlattices. However, the preformed GNP arrays cannot selectively place the target sample at the “hotspot” specifically to obtain the highly enhanced signal, therefore, different approaches have been reported for locating the target molecule at the hotspot.

Several studies have recently reported the development of a SERS-based biosensor that acts as a “direct biosensor” by detecting specific signals from the target biomolecules, such as neurotransmitters [38], nucleosides [39], and microRNA (miRNA) [40]. Tu and his coworkers developed a SERS-based serotonin analysis method by using GNPs in the presence of various closely related precursors and metabolites (Figure 2B) [38]. Due to their structural similarity, serotonin and tryptophan—a precursor of serotonin—show very similar SERS spectra when mixed with GNPs. To distinguish two amino acids, SERS measurements were conducted under a range of different pH conditions covering the p*K*_1_ value. Particularly, the SERS signal intensity of serotonin was significantly higher than tryptophan at pH 5.1 and pH 4.1 conditions. This is likely a result of GNPs aggregating with the positively charged ampholytes, forming 3D complexes. Similarly, Feng and his coworkers analyzed the differences of nucleosides between nasopharyngeal and esophageal cancers collected from the urine by SERS after incubating with GNPs [39].

By taking advantage of the chemical-specific spectral information in Raman spectroscopy, the combination of distinct Raman frequencies from different chemicals is able to be utilized as a barcoding system, as well as an “indirect biosensor” [41]. This Raman barcoding system provides the potential to overcome a “multiplexing ceiling” from existing optical materials. Cho and his coworkers developed a Raman barcoding-based biosensor with GNPs to diagnose and analyze circulating cancer stem cells (CCSCs) in the presence of circulating tumor cells (CTCs) (Figure 2C) [42]. For this approach, five different combinations of Raman-active nanoprobes (RANs) were prepared, each consisting of a GNP (as a Raman signal enhancer), aromatic thiol (as a Raman reporter), antibodies, and thiolated DNA (as a capturing linker). When the CCSCs and the CTCs with spiked with five families of RANs in the blood, the subtype-specific SERS signals decorated the cell and were analyzed once the cell was captured by the microfluidic device. Another way to develop an “indirect biosensor” is by combining GNPs’ SERS and catalytic properties to provide a particularly promising platform for the real-time probing of GNP-catalyzed reactions and for developing sensitive bioassays. Hu and her coworkers synthesized a nanomaterial which embedded GNPs and glucose oxidases in the metal–organic framework to generate hydroperoxide. The generated hydroperoxide induced the activation of a co-delivered precursor (leucomalachite green, LMG) into the active form (malachite green, MG) (Figure 2D) [43]. The assembled oxidase oxidized the substrate (i.e., glucose or lactate), and the hydroperoxide was produced based on the amount of the substrate. The generated hydroperoxide continued to oxidize LMG to the Raman-active MG via the GNPs’ catalysis, as measured by SERS. After optimizing the in vitro sensing performance, it was further employed to monitor the change of glucose and lactate in living brains following the ischemia/reperfusion model. Even though SERS-based biosensors using GNPs have shown great performance for the detection and analysis of target molecules, some improvements are needed to overcome current limitations, including signal reproducibility and sensitivity.

## 4. Gold Nanoparticle-Based Plasmon-Enhanced Fluorescence and Quenching Biosensor

Plasmonic GNPs have another interesting optical phenomenon with localized fluorophores, which is highly useful for the development of biosensors [44]. In general, GNP can lead to both fluorophore quenching and enhancement that can be explained by a well-established Förster (or fluorescence) resonance energy transfer (FRET) mechanism [45,46]. When the GNP and the fluorophore are within a few nanometers from each other, the non-radiative local field of one material (donor) can excite the other one (acceptor), which then translates into either the quenching or enhancement of the fluorescent signal. More specifically, the quenching happens when FRET occurs from the fluorophore to GNP; alternatively, the enhancement of fluorophore, which is typically referred to as plasmon-enhanced fluorescence (PEF), can happen, depending on the electromagnetic field generated from the plasmonic metal surface [16]. Particularly, it is well known that these effects can be balanced by the spatial variation (distance) between the GNP and the fluorophore [10,47]. For example, fluorophore quenching can be observed at a short distance (<5 nm) and enhanced fluorescence can be observed at an adjusted distance (~10 nm). It should be noted that GNPs can also enhance the radiative rate of the fluorophore through the Purcell effect at longer distances (10~50 nm); however, since the resulting Purcell effect is negligible between plasmonic metals and fluorophores, it will not be discussed herein [48].

**Figure 3:**
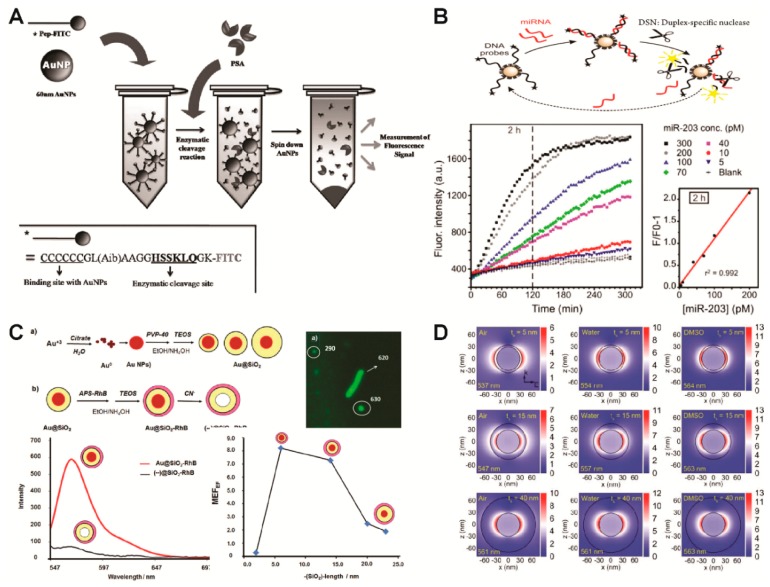
Plasmonic biosensor based on plasmon-enhanced fluorescence and quenching. (**A**,**B**) Molecular detection based on fluorescence by utilizing the gold nanoparticle as quenching material; (**C**) development of highly fluorescent core/shell nanoparticles by utilizing gold nanoparticle as core for detection of bacteria; (**D**) simulation study on plasmon-enhanced fluorescence (PEF) based on shell thickness to optimize fluorescence enhancement. (**A**) Figure reproduced with permission from [49], © 2013 Elsevier; (**B**) Figure reproduced with permission from [44], © 2014 American Chemical Society; (**C**) Figure reproduced with permission from [45], © 2017 Royal Society of Chemistry; (**D**) Figure reproduced with permission from [47], © 2017 American Chemical Society.

Oh and his coworkers have developed a fluorescent biosensor based on a site-specific enzymatic cleavage reaction to detect a prostate-specific antigen (PSA) by utilizing the GNPs as energy acceptors (quencher) (Figure 3A) [49]. A small peptide sequence with a fluorophore (fluorescein isothiocyanate, FITC) on one end was conjugated to the GNP through the functional amine group on the other end. Initially, fluorescence associated with the fluorophore was quenched due to the FRET between the fluorophores and the GNPs; however, when the PSA recognized and cleaved the specific sequence of the peptides conjugated to the GNP, a recovery of fluorescence signal was observed due to the splitting of the FITCs from the GNP. In a similar way, Degliangeli et al. functionalized GNPs with fluorophore-labeled DNA-probe strands to detect miRNA (Figure 3B) [50]. The presence of target miRNA led to the hybridization of DNA–RNA strands, and, following enzyme hydrolysis, fluorescence signal recovery was observed by splitting fluorophores from GNPs.

As an alternative direction for developing fluorescent based biosensors, Bracamonte and his group have developed highly fluorescent nanoparticles by utilizing GNPs as the core, silica as the shell (as a spacer), and rhodamine b (RhB) as a fluorophore for bacteria imaging and sensing (Figure 3C) [51]. The plasmon resonance maximum of the core–shell nanoparticles was able to be red shifted due to interactions with the GNP core based on the thickness of the silica shell. The PEF effect from the GNP core was also evaluated by applying sodium cyanide as a gold leeching material. In addition, bright and clear bacteria images were obtained by laser fluorescence microscopy after labeling them with the developed core/shell PEF nanoparticles. Teixeira et al. also utilized GNPs as cores and covered them with an polyelectrolyte shell to promote enhancements for a phthalocyanine fluorescent dye [52]. Similar to these approaches, various studies were performed to verify the effect of spatial variation by controlling spacing within shell thickness. For example, Montaño-Priede et al. demonstrated that the maximum fluorescence enhancement of quantum dots (QDs, CdSe) was observed when a silica shell between a GNP core and QDs was approximately 10 nm; however, both the reduction and the increase of shell thickness diminished the enhancement effect of the fluorescence either by energy transfer from the QDs to GNP or a larger separation between the CdSe QDs and GNP core, respectively (Figure 3D) [53].

## 5. Gold Nanoparticle-Based Colorimetric Biosensors

Similar to the aforementioned unique optical properties of GNPs, the color of the GNP solution is also affected by the LSPR phenomenon upon the aggregation and dispersion of the GNPs [54]. The aggregation of GNPs can induce the coupling of the plasmon modes when the interparticle distances are less than 2.5 times their diameters [55]. This electromagnetic coupling of GNPs induced a red shift and broadening of the plasmon resonance peak, associated with the longitudinal resonance [56]. The aggregation or dispersion of the GNPs is influenced by changes to the external environment, including pH [57], chemicals [17], metal ions [58], and biomolecules (DNA [59,60,61], miRNA [62], peptide [63], and proteins [64,65]).

**Figure 4:**
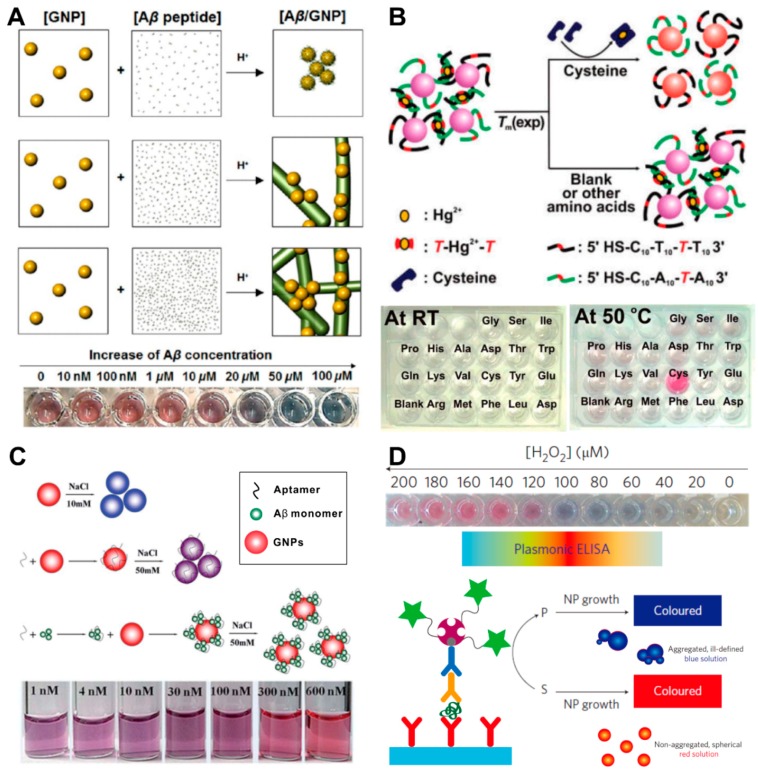
Gold nanoparticle-based colorimetric biosensors. (**A**) Gold nanoparticles as a nucleation core and colorimetric optical reporters for tracking amyloid-beta; (**B**) cysteine-specific colorimetric sensor based on dispersion of gold nanoparticle aggregate with Hg^2+^-complexed T–T mismatched duplex DNA; (**C**) aptamer analysis of DNA length and hybridization kinetics based on dimerization of gold nanoparticles; (**D**) detection of mRNA splice variants in live cells based on dimerization of gold nanoparticles. (**A**) Figure reproduced with permission from [63], © 2013 American Chemical Society; (**B**) Figure reproduced with permission from [60], © 2008 American Chemical Society; (**C**) Figure reproduced with permission from [71], © 2018 Royal Society of Chemistry; (**D**) Figure reproduced with permission from [17], © 2012 Nature Publishing Group.

Depending on the interaction between targets and GNPs, GNPs can have roles as effective nucleation sites for fast-catalyzing the target aggregation. Choi and his coworkers utilized GNPs as a nucleation core and colorimetric optical reporters for tracking amyloid-beta (Aβ) aggregation (Figure 4A) [63]. The Aβ monomers were electrostatically attached to the surface of the GNPs at pH 2~3, since Aβ has a positive charge while GNPs have a negatively charged surface. The aggregation speed and size of the aggregated composites are directly related to the concentration of Aβ in the sample, and it can be monitored by a color change, from red to purple or blue. This colorimetric biosensing platform was also used to confirm the inhibition properties of anti-Aβ antibodies and human serum albumin on the Aβ oligomerization. Similarly, Lou and her coworkers found that digested DNA has superior abilities over intact DNA for binding to unmodified GNP solution at high salt concentration [66]. However, these non-crosslinking aggregation-based colorimetric biosensors show several limitations in signal stability, reproducibility, and target specificity because of the nonselective interaction between the target and the GNPs and lack of a crosslinking method to overcome the interparticle repulsive forces.

To induce target-specific GNP aggregation, GNPs have been functionalized with target-recognizing molecules, such as single-stranded DNA (ssDNA) [59,60], antibodies [67], and aptamers [68,69,70,71]. After Mirkin and his coworkers reported a biosensor in 1997 which can analyze a single mismatch in the hybridized DNA by using the ssDNA-conjugated GNPs [59], DNA and RNA samples have been analyzed with an interparticle crosslinking aggregation-based colorimetry method [54]. Lee and his coworkers designed GNP networks interconnected with duplex DNA with strategically placed Hg^2+^-complexed T–T mismatches (Figure 4B) [60]. The cysteine analyte can bind mercuric ion and remove it from the aggregate, thereby lowering the DNA dissociation temperature. In this way, the presence of cysteine can be detected in a specific manner. Similarly, aptamer-modified GNP-based colorimetric biosensors have been developed to detect proteins [67], peptides [71], and bacteria [72]. Zhu and his coworkers showed the different degrees of GNP aggregation profiles when comparing the GNP solution containing aptamer only with the GNP containing an Aβ–aptamer with sodium ions (Figure 4C) [71]. The Aβ–aptamer complexes were stabilized by the GNPs in the presence of sodium ions in the colloidal solution.

In the case of colorimetric biosensor-based protein detection, GNPs are also applicable for the detection of enzymatic reactions [17,73]. De la Rica and his coworkers developed a plasmonic enzyme-linked immunosorbent assay (ELISA) as a colorimetric biosensor (Figure 4D) [17]. In the presence of the analyte, the enzyme catalase consumes hydrogen peroxide, and low concentrations of hydrogen peroxide favor the formation of aggregated, spherical nanoparticles that give rise to a blue solution.

## 6. Conclusions and Future Outlook

Recent advances in both nanofabrication/nanochemistry and detection techniques have led to multiple approaches for the research and development of plasmonic biosensors. The unique plasmonic properties of GNPs provide enormous potential for biosensing research when they are combined with a material, having such advantages as easy preparation, biocompatibility, inertness, and intense light emissions. A large variety of designs of GNP-based systems have been reported to improve their plasmonic properties in the specific optical systems for the targeted applications. For example, (1) immobilizing or patterning GNPs on the surface of a metal substrate for an LSPR-based biosensor; (2) controlling the interparticle gap distance of GNPs to generate “hotspots” for the SERS-based biosensor; (3) controlling the distance between a fluorophore and a GNP for enhancing or quenching the fluorescent signal; and (4) controlling the dispersion stability in a solution for construction of a colorimetric biosensor (Table 1).

The performance of GNP-based plasmonic biosensors shows clear advantages when compared to other optical biosensors; however, a lack of signal reproducibility, standardization, and the necessity of confirmation with conventional methods forms a hurdle for the plasmonic biosensors to be fully implemented in practice when answering significant biomedical questions. The major concern for plasmonic biosensors is their signal reproducibility, because randomly conjugated analytes or randomly aggregated GNPs can generate highly enhanced signals that do not match actual conditions. Homogeneously nanopatterned substrates can help induce LSPR, generate the “hotspot” uniformly, and guide the analyte into the “hotspot” specifically. Moreover, analyzing the sample by combining different sensing techniques can provide new and precise information for specific analytes. For example, the chemical structure of an analyte can be changed by oxidation/reduction, therefore, spectroelectrochemical biosensors [74]—combining plasmonic signal detection and electrochemical detection techniques—can be one of the ways to break through current limitations (i.e., difficulty distinguishing similarly structured chemicals) of plasmonic biosensors. Recently, Kim and his coworkers reported a potential spectroelectrochemical signal detection platform—the large-scale homogeneous nanoelectrode array—which shows a homogeneous SERS signal [75].

Although the instability of the biological materials (e.g., DNA, enzyme, aptamer, etc.) is still one of the major limiting factors for the development and commercialization of the biosensors, many studies have been performed to overcome this issue through the development of chemical-based biomolecules. For example, DNA-binding hairpin pyrrole-imidazole (Py-Im) polyamide—a class of heterocyclic amino acid oligomers—was conjugated on the GNP for DNA detection instead of the DNA-binding protein [76]. Peptide nucleic acid (PNA) is also used to improve stability by substituting nucleic acid (DNA/RNA) or aptamers [77]. In addition, the proper immobilization of enzymes tends to improve stability. Some immobilization techniques can diminish the activity of the enzyme due to random orientation or denaturation of the enzyme; however, proper immobilization approaches are found to be effective at maintaining enzyme activity, as well as biosensor stability [78,79].

Consequently, in combination with these newly developed methods, we believe with the further improvement of sensitivity, selectivity, and reproducibility of GNP-based plasmonic biosensors, plasmonic biosensors will find more impactful applications in biological, biochemical, and medical fields.

## Figures and Tables

**Table 1:** Gold nanoparticle-based plasmonic biosensors.

Plasmonic Biosensors	Advantages	Limitations	Sensitivity	Ref
Localized Surface Plasmon Resonance	Ease of operation Possibility of high-throughput sensing	Not able to distinguish different binding events regarding multiple analytes Detection limits with higher resolution or better signal to noise ratio	>pM	[11,25,26,27,80]
Surface-Enhanced Raman Spectroscopy	High specificity Available for fingerprinting	Low signal reproducibility	>aM	[38,43,81]
Plasmon-Enhanced Fluorescence and Quenching	Increased detection sensitivity Protection from photobleaching	Difficulty in the precise and cost-effective fabrication of metallic nanostructures Difficulty in selective functionalization in plasmonic hotspots	>fM	[49,50,51,53,82]
Colorimetry	Monitoring with naked eyes Fast reaction	Difficulty in quantitative analysis in a high-resolution manner	>pM (aggregation) >aM (Enzymatic reaction)	[17,54,64,65,67,73]

## Abbreviations

GNPs	gold nanoparticles
LSPR	localized surface plasmon resonance
SERS	surface-enhanced Raman scattering
NIR	near-infrared
HIV	human immunodeficiency virus
DNA	deoxyribonucleic acid
RNA	ribonucleic acid
FRET	Förster (or fluorescence) resonance energy transfer
PEF	plasmon-enhanced fluorescent
PSA	prostate-specific antigen
FITC	fluorescein isothiocyanate
QDs	quantum dots
CCSCs	circulating cancer stem cells
CTCs	circulating tumor cells
RANs	Raman-active nanoprobes
miRNA	micro RNA
LMG	leucomalachite green
MG	malachite green
Aβ	amyloid beta
ssDNA	single-stranded DNA
ELISA	enzyme-linked immunosorbent assay
